# Responsible pricing in value-based assessment of cancer drugs: real-world data are an inevitable addition to select meaningful new cancer treatments

**DOI:** 10.3332/ecancer.2017.ed71

**Published:** 2017-09-11

**Authors:** Wim van Harten, Maarten J IJzerman

**Affiliations:** 1 The Netherlands Cancer Institute, Plesmanlaan 121, 1066 CX Amsterdam,The Netherlands; 2 Health Technology and Services Research (HTSR), University Twente, Drienerlolaan 5, 7522 NB Enschede, The Netherlands; 3 Cancer Economics Working Group, European Organization of Cancer Institutes, 11 Rue d’Egmont, B-1000, Brussels, Belgium; 4 Rijnstate Hospital, Wagnerlaan 55, 6815 AD Arnhem, The Netherlands; 5 Luxembourg Institute of Health, Health Economics and Personalized Medicine, 1A-B, rue Thomas Edison, L-1445 Strassen, Luxembourg

**Keywords:** Cancer Drugs, Cost Effectiveness, Technology Assessment, Data

## Abstract

Recently, NICE was given the task of governing the Cancer Drug Fund (CDF) in the UK as the latter was criticized for allowing too many insufficiently tested drugs to be covered [1, 2]. The CDF was initiated in 2012, but immediately received criticism from several health economists because of the rather strict coverage criteria that are commonly used by NICE for most other health services in the NHS. This led to questions about the use of different reimbursement criteria (why have a different fund otherwise?) for expensive cancer drugs. Such a separate fund would potentially take away large amounts of the collective health budget. This led to questions about the use of different reimbursement criteria (why have a different fund otherwise?) for expensive cancer drugs compared to other technologies. This is just one example of discussions that are taking place in many countries on the issue of drug coverage policies.

This development takes place against a background of increasingly intense discussion on pricing and affordability of (new) cancer drugs, the responsible behavior of pharmaceutical companies that spend public resources for R&D, and the lack of transparency in pricing and R&D expenditure in combination with profit margins of sometimes up to 20%. We argue that Real-World Evidence (RWE) may play a much greater and, on occasion, pivotal role in developing sustainable cancer care, because it allows much better estimates of actual drug use and costs and increases transparency in health outcomes.

## Cancer Drug Fund and coverage with evidence development

While the CDF was clearly initiated to provide early access to drugs for patients and their physicians, there is now general agreement that the CDF is not sustainable and requires a major reform. In addition, even a large survey amongst the general public concludes that the government should not give priority to a separate CDF [3]. As several other countries have experience with coverage - with evidence development, the reform of the CDF will now give NICE the challenge to implement a kind of Coverage with Evidence Development (CED) scheme, in which evidence for full reimbursement of drugs can be collected, while the drug is used as a temporary measure. The concept of CED or performance-based risk-sharing arrangements originated in the US and is increasingly used in many countries as it ensures early access to promising treatment innovations while the lengthy process of filing for reimbursement is not yet completed, lacks certain data or needs a longer follow up using real-world data. CED schemes are used to grant early access to promising innovative technologies, while the evidence (or coverage admission file) is insufficient and research, especially aspects of technology assessment or health economic evaluation, is continued while coverage is temporarily allowed during this period. An advantage of CED is its use in less strict selected populations producing realistic evidence on indications in practice (versus the often very selected trial populations) and a wider range of HTA relevant aspects as well as real-world data [4–6]. Disadvantages are the sometimes less strict methodological conditions, such as cohort-based patient registries for which long term follow up is sometimes troublesome and outcome criteria may change over time. It is thus not always easy to base firm conclusions on this material.

## The need for real-world data

In addition to the early access to cancer drugs, there are other reasons for implementing a performance-based risk sharing agreement, which is the use of real-world data to generate a more realistic evidence base. Recently, Peter Wise (2016) reviewed the way cancer drug trials are being performed in a BMJ paper in response to criticisms of the real-world effectiveness of cancer drugs [7]. Apart from the need to appropriately inform patients through shared decision making, he also points at the circumstances that trials are conducted in. Due to various (and sometimes legal) reasons, populations included in trials and the delivery of health services differ considerably from the real-world situation.

Using real-world data in so called “patient registries” or “clinical audit systems” can, however, be a very powerful instrument in improving health care practice. In particular, the recent criticisms of the lack of – or at least disappointing effectiveness of - cancer drugs in the real world, suggest that trials overestimate the effects and thus emphasize the need not to be satisfied with trial data that originate from selected populations under strictly controlled circumstances. In many types of surgical oncology, and increasingly medical oncology and radiotherapy, registries are used to follow patients to quantify and reduce complication rates, reduce harm and improve effectiveness [8, 9]. Especially in view of this, selecting certain groups of patients or treatment populations for CED with prolonged follow up and continuous registration can be very useful. Of course, endpoints have to be defined, registration comes at a price (although 12 courses of Nivolumab for a single patient will cover the registration costs for all cancer patients in the Netherlands) and these data are hard to compare to original trial sets because of the lack of methodological validity. And obviously, the registration is only partly controlled by Pharma as different stakeholders are involved.

In the Netherlands, the concept of patient registries is also used for the introduction of innovative cancer drugs and trial-based CED schedules are applied in, for example, immunotherapy through Tumour Infiltrating Lymphocyte treatment (an expensive cell therapy-based treatment of around € 65.000 per patient) in advanced melanoma patients.

## Real-world data and pricing

Data are the essential factor in cost-effectiveness analysis, influencing both the nominator (the costs), and the denominator (QALYs). QALYs are part of many pharmaco-economic guidelines, yet criticism is possible of their use in different age categories, end of life care and between different types of disease, and the inability to incorporate process and experience with healthcare delivery.

In economic evaluations, the common guideline is to use the official list prices of expensive drugs. List prices are publicly available and generally comparable across countries, but are different from actual prices hospitals pay for drugs. A recent overview of listed and actual drug costs in Europe showed that rebates are given differently per- and between countries, leading to prices that can differ up to 100% between countries and are sometimes up to 30% lower than list prices [10]. This calls for an approach where real-world prices are transparent and can be used to inform the discussion on acceptable price levels in relation to the affordability per country. Very recently, Hawkes reported on a number of drugs being (significantly) lowered in price and accepted by NICE in a very short period; which is explained by the fact that transparency down-regulates prices [11]. In addition, it seems obvious that the use of actual prices including rebates will result in much more favorable cost-effectiveness ratios, which must be significant in view of the thresholds being used in other western countries. Apart from that, CED data and data from patient registries are an important addition to feed those analyses with real-world data.

## Responsible pricing

Another and more recent discussion on value-based pricing refers to the (de-)linkage of value and costs. The incremental cost-effectiveness ratio combines the incremental costs to the benefits, and thus creates a mechanism that new technologies will be reimbursed at a cost that would fall around the willingness-to-pay threshold. There are good reasons for this approach, but the downside is that there is not a mechanism to link sales prices to actual development costs or “value”. In pharmaceutical marketing, value-based pricing lacks clear definitions and it seems rather that given a certain value the maximum possible price is negotiated from a certain market. Other views have been expressed in Value-Based Healthcare, suggesting that healthcare be organized so as to generate the highest value at the lowest costs. Also, the World Health Organization (WHO) in a recent meeting in the Hague at the fair pricing forum, suggested that value should be de-linked from what we are willing to pay for a new drug. This idea is not new, yet does not completely solve the problem. Nevertheless and in view of a clear consensus, it is fair to conclude that societies have to be responsive towards the actual prices of cancer drugs in relation to their development cost. This is even more so the case with costly public investments in commercial R&D, through several public-private partnerships with the objective to retain influence on pricing decisions.

## Conclusions

From a public health point of view there is great need for responsible pricing through transparency, innovative drug development and licensing schedules and responsible behavior by Pharma. Continuous pressure on these domains is needed to obtain pricing levels that no longer compromise the financial sustainability of the cancer system in the long run.

Real-world data are a valuable additional data source in coverage decisions. Flaws in validity due to organizational issues and missing data or length of follow up should not be a reason to discard them but rather to strive for improved real-world effectiveness data. This influences the CEA equations by redefining the effectiveness score as part of the denominator with population-based data. Our presumption is that we need both traditional trial-based- as well as real-world- or patient registry data.

The present price levels of drugs seem not to be sustainable in view of the promising pipelines. Looking at recent developments in drug pricing and coverage decisions in the UK but also other countries, agencies are findings ways to improve the cost effectiveness (value) of cancer drugs by further clarifying the denominator (data) and especially influencing the nominator (costs).
